# A subset of patients with systemic lupus erythematosus fails to degrade DNA from multiple clinically relevant sources

**DOI:** 10.1186/s13075-015-0726-y

**Published:** 2015-08-13

**Authors:** Jonatan Leffler, Katarzyna Ciacma, Birgitta Gullstrand, Anders A. Bengtsson, Myriam Martin, Anna M. Blom

**Affiliations:** Lund University, Department of Translational Medicine, Section of Medical Protein Chemistry, Inga Marie Nilssons gata 53 floor 4, 205 02 Malmö, Sweden; Telethon Kids Institute, University of Western Australia, 100 Roberts Rd, Subiaco, WA6008 Western Australia Australia; Lund University, Department of Clinical Sciences, Section of Rheumatology, Skåne University Hospital Lund, 221 85 Lund, Sweden

## Abstract

**Introduction:**

Patients with systemic lupus erythematosus (SLE) have a decreased ability to clear cell remnants and multiple deficiencies in the ability to degrade cellular chromatin have been linked to the disease. Since the discovery of neutrophil extracellular traps (NETs), a renewed interest has been sparked in this field of research with multiple studies reporting a decreased ability of patients with SLE to degrade NETs. In this study we extend these findings by investigating the ability of patients with SLE to degrade chromatin from multiple clinically relevant sources.

**Methods:**

We use flow cytometry in combination with NET degradation and DNA zymogram assays to investigate the ability of sera from SLE patients to degrade chromatin from three different sources of DNA such as NETs, apoptotic and necrotic cells. This ability was further associated with clinical manifestations.

**Results:**

We found that 61 % of the patients had an affected degradation of at least one chromatin source. Further, degradation of NETs correlated with degradation of chromatin from secondary necrotic cells but not with degradation of chromatin from primary necrotic cells. Patients who fail to degrade several forms of DNA more often display anti-nuclear and nephritic involvement whereas this is not observed in patients with decreased ability to degrade chromatin from primary necrotic cells.

**Conclusions:**

The majority of patients with SLE has a decreased ability to degrade chromatin from clinically relevant sources. This decreased ability is further reflected in their clinical presentation.

## Introduction

Patients with the autoimmune disorder systemic lupus erythematosus (SLE) exhibit, for yet non-clarified reasons, a decreased ability to degrade DNA. The phenomenon was first observed in the 1960s [1], and has recently regained new interest with the discovery of neutrophil extracellular traps (NETs) [2]. NETs consist of chromatin covered with antimicrobial proteins and constitute a candidate autoantigen target in SLE. SLE is characterized by an autoimmune reaction against many nuclear antigens historically proposed to originate from apoptotic cells that are not properly cleared [3]. DNA in various forms is degraded by specific nucleases where DNase-I is the main enzyme responsible for degrading DNA and chromatin released into serum. The role of DNase-I in SLE received attention when it was discovered that the presence of a DNase-I inhibitor correlated with levels of nuclear autoantibodies [1] and that SLE patients had decreased serum nuclease activity [4]. Attempts were subsequently made to restore the activity by infusion of recombinant DNase-I but without reaching a sufficient serum concentration to lead to clinical improvements [5]. Recently, it was confirmed that sera from a subgroup of SLE patients do not degrade NETs [6, 7].

Although NETs pose a highly interesting target in SLE, other chromatin sources like apoptotic and necrotic cells should not be forgotten. The importance of serum nucleases for the degradation of apoptotic and necrotic cells has been studied previously and it appears that this process is dependent on additional cofactors, such as complement C1q [8], serum amyloid P [9], factor VII-activating protease [10] and plasmin [11] - at least for degradation of necrotic chromatin. These cofactors are thought to open the normally condensed chromatin structure by displacing histone H1. NETs consist of decondensed, open chromatin and therefore degradation is not dependent on such cofactors. In our previous study we instead observed that C1q inhibited degradation of NETs and hypothesized this to be a trade-off for opsonisation [6], which was later confirmed by another group [12]. The current literature hence proposes that multiple factors are important in the degradation of DNA depending on the source and nature of the chromatin. Patients with SLE have been described to have a decreased DNase activity [4] but how that relates to different forms of DNA is still unclear. We therefore set out to investigate how patients with SLE degrade DNA from a range of clinically relevant sources. The aim was to generate a more comprehensive image of DNA degradation in SLE and determine what sources of DNA most likely are involved in disease pathology. In the study, we focused on DNA sources with known serum nuclease-dependant degradation and used DNA in the form of NETs as well as chromatin from both primary and secondary necrotic cells and compared to degradation of purified DNA using a zymographic approach. The results additionally led us into some fundamental characterization of the interactions between DNase-I and serum proteins.

## Methods

### Patients and sera

A total of 66 SLE patients (5 men and 61 women) with a median age of 39 years (range 18–75), fulfilling at least four American College of Rheumatology (ACR) 1982 classification criteria for SLE [13] were recruited at the Clinic of Rheumatology, Skåne University Hospital in Lund (Sweden). The distribution of ACR classification criteria for SLE is described in Table 1. Disease activity was recorded using the SLE disease activity index 2000 (SLEDAI-2K) [14]. Sera from 103 healthy volunteers with matched age and sex were used as controls in the study. All patients and healthy controls gave informed consent to participate in the study, which was approved by the local ethics committee (Lund University) according to the Helsinki declaration.

**Table 1:** Patient characteristics according to American College of Rheumatology (ACR) criteria

ACR criteria	Number of patients (%)
Malar rash	44 (66.7)
Discoid rash	18 (27.3)
Photosensitivity	42 (63.6)
Oral ulcer	16 (24.2)
Arthritis	53 (80.3)
Serositis	35 (53.0)
Nephritis	32 (48.5)
Neurological disorder	3 (4.5)
Hematological disorder	37 (56.1)
Immunological disorder	51 (77.3)
Anti-nuclear antibodies	66 (100)

### Degradation of cell chromatin

Jurkat T-cells (ATCC, Manassas, VA, USA) were cultured in Roswell Park Memorial Institute medium (RPMI) with 10 % foetal calf serum at 37 °C and 5 % CO_2_. For experiments, the cells were washed twice, kept in RPMI and either used directly as live cells alternatively rendered apoptotic, primary or secondary necrotic. Apoptosis was induced by incubation with 1 μM staurosporine (Sigma-Aldrich, St Louis, MO, USA) at 37 °C for 3 h. Primary necrosis was induced through incubation in 15 % EtOH at 37 °C for 1 h and secondary necrosis was induced with 20 μM oxaliplatin (Teva, Petach Tikva, Israel) at 37 °C for 48 h as previously described [15]. The cell states were confirmed with Annexin V (Immunotools, Friesoythe, Germany) and Via-Probe (BD, San Jose, CA, USA) staining. For degradation experiments, cells were washed and incubated with 1.5 % (for degradation of primary necrotic chromatin) or 3 % (for degradation of secondary necrotic chromatin) patient sera, pooled normal human serum (NHS) or fractionated sera for 3 h at 37 °C in 10 mM Tris-HCl, pH 7.5, 50 mM NaCl, 10 mM MgCl_2_ and 2 mM CaCl_2_ (DNase buffer). To analyze DNA degradation, we used Hoechst (Invitrogen, Waltham, MA, USA) or Via-Probe to detect cellular double stranded DNA (dsDNA). Loss of signal indicates nearly complete degradation to small fragments (below 20 bp). DNA content was analyzed in a CyFlow Space (Partec, Görlitz, Germany) flow cytometer.

### Isolation of neutrophils

Neutrophils were isolated from healthy volunteers according to a previously published method [16]. Briefly, blood from healthy volunteers was separated by centrifugation on a Histopaque 1119 column (Sigma-Aldrich), the granulocyte-rich fraction was isolated and washed, and neutrophils were isolated by centrifugation on a Percoll gradient (65 − 80 %) (GE-healthcare, Fairfield, CT, USA) and isolated from the intersection of the 70 % and 75 % layer, washed and resuspended in RPMI with 10 mM Hepes. Purity of neutrophils (>80 %) was determined by surface marker expression for anti-CD14 (BD), anti-CD15 and anti-CD16 (both from Immunotools) and defined as CD16^+^/CD15^+^/CD14^low^.

### Generation and degradation of NETs

Freshly isolated neutrophils, 50,000/sample, were seeded onto a 96-well flat-bottom plate (Nunc, Waltham, MA, USA) with 20 nM PMA (Sigma-Aldrich) for 4 h at 37 °C and 5 % CO_2_ to generate NETs. After incubation, cell medium was removed and 10 % patient sera, control sera or fractionated sera in DNase buffer was added and incubated for 60 minutes at 37 °C. During this time, degraded NETs were released into solution. Aliquots of the solution containing NETs were then transferred to PBS with a final concentration of 2 mM EDTA to stop further degradation and DNA content was quantified using PicoGreen (Invitrogen). As the internal control, pooled NHS was used and all samples were compared to the mean of the internal controls for each individual experiment. All samples were measured twice, first in duplicates followed by once in singles and the mean of the two measurements was used for analysis.

### Gel filtration

Serum diluted to 50 % with or without 500,000 cpm = counts per minute ^125^I DNase-I (Bioworld, Dublin, OH, USA), labeled using the chloramine T method, was diluted in DNase buffer and separated on a Superose 12 column (GE healthcare) using the ÄKTA system (GE healthcare). The column was washed with DNase buffer and 0.1 ml fractions were collected. For assays using radiolabeled DNase-I, radioactivity was measured in fractions before stored at −80 °C until further used.

### DNase zymogram

Sera from SLE patients and controls were diluted in DNase buffer to 30 % and loaded onto a native PAGE gel containing 22 μg/ml denatured calf thymus DNA (Sigma-Aldrich). As internal controls 30 % NHS was used. Gels were run at 90 V for 2 h under native non-reducing conditions, washed with dH_2_O and incubated with 40 mM Tris–HCl pH 7.5, 8 mM MgCl_2_, 2 mM CaCl_2_, 0.02 % NaN_3_ at 37 °C for 18 h. After this, ethidium bromide (EtBr) was added to a final concentration of 1 μg/ml and gels were incubated at 37 °C for an additional 30 minutes and analyzed in a ChemiDoc™ MP Imaging System (BioRad, Hercules, CA, USA). For complex formation assays, 2.3 μg/ml AF488-labeled DNase-I was incubated with 5–200 μg/ml actin from rabbit skeletal muscle (Sigma-Aldrich) or 150–200 μg/ml Gc-globulin (Sigma-Aldrich) in 10 % NHS or heat-inactivated NHS (Hi-NHS) and run as above.

### Statistical analyses

For comparison of chromatin degradation between two groups the Mann-Whitney test was used. For comparison between multiple experimental parameters two-way analysis of variance (ANOVA) was used followed by Bonferroni post hoc test in GraphPad Prism 5 (GraphPad, La Jolla, CA, USA). Association with disease manifestation was tested using Pearson’s chi-square (χ^2^) test. For principal component analysis, cluster analysis and analysis of association with disease manifestation was performed using JMP 11 (SAS, Cary, NC, USA).

## Results

### Chromatin degradation in primary and secondary necrotic cells is dependent on serum nucleases

**Fig. 1:**
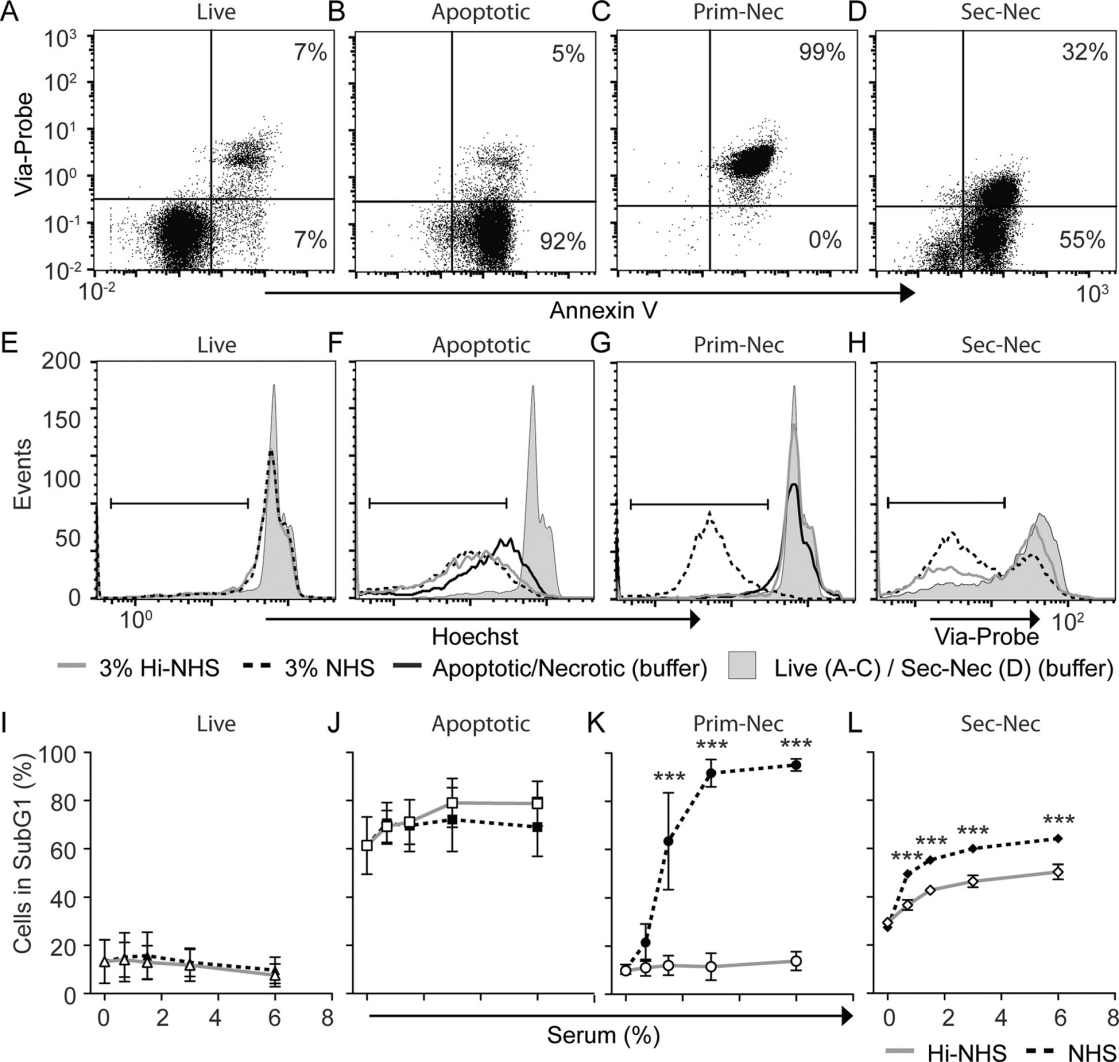
Serum nucleases degrade chromatin in primary and secondary necrotic cells. Annexin V and Via-Probe was used to define live (**a**), apoptotic (**b**) primary necrotic (*Prim-Nec*) (**c**) and secondary necrotic (*Sec-Nec*) (**d**) cell states in Jurkat T-cells. The degradation of chromatin in live (**e**), apoptotic (**f**) primary (**g**) and secondary necrotic (**h**) cellular DNA content was determined after incubating cells with normal human serum (*NHS*) or heat-inactivated normal human serum (*Hi-NHS*). Cells with subnormal DNA content as indicated by the vertical gate were quantified for different serum concentrations and displayed as percentage of the total cell population (**i-l**). **a**-**h** Representative histograms of three independent experiments using 3 % serum in **e**-**h. i**-**l** Mean ± SD of **e**-**h** at varying serum concentrations. Significance of difference was calculated using two-way analysis of variance with the Bonferroni post hoc test; ***p <0.001

To analyze degradation of chromatin in dying cells we induced different forms of cell death and analyzed cell states using Annexin V and Via-Probe (Fig. 1a-d). Annexin V detects the externalization of phosphatidylserine, which is initiated early in dying cells, whereas Via-Probe binds DNA in cells that have lost membrane integrity during later stages of cell death. Live cells are hence double negative (Fig. 1a), apoptotic cells are only positive for Annexin V (Fig. 1b), primary necrotic cells are double positive (Fig. 1c) and secondary necrotic cells display a mixed population (Fig. 1d). To compare the need for active serum nuclease for degradation of chromatin in these forms of cell death, cellular DNA content was measured by flow cytometry after incubation with either NHS or Hi-NHS. As expected, no degradation of chromatin was observed when live cells were incubated with NHS or Hi-NHS (Fig. 1e and i). While the majority of apoptotic cells endogenously degraded chromatin, there was no additional degradation by NHS compared to Hi-NHS (Fig. 1f and j). Unlike apoptotic cells, no endogenous nuclease activity appeared to be activated in primary necrotic cells and chromatin was only degraded when incubated with NHS but not with Hi-NHS (Fig. 1g and k). We also observed endogenous degradation in secondary necrotic cells, however there was also serum-dependent degradation (Fig. 1h and l). Thus, we can conclude that apoptotic chromatin is initially degraded by endogenous nucleases, primary necrotic cells by serum nucleases while secondary necrotic cells are degraded by both endogenous and serum nucleases. As our aim was to investigate the role of serum nucleases in SLE, we focused the study on the degradation of primary and secondary necrotic cells.

### Degradation of chromatin from multiple sources is decreased in SLE

**Fig. 2:**
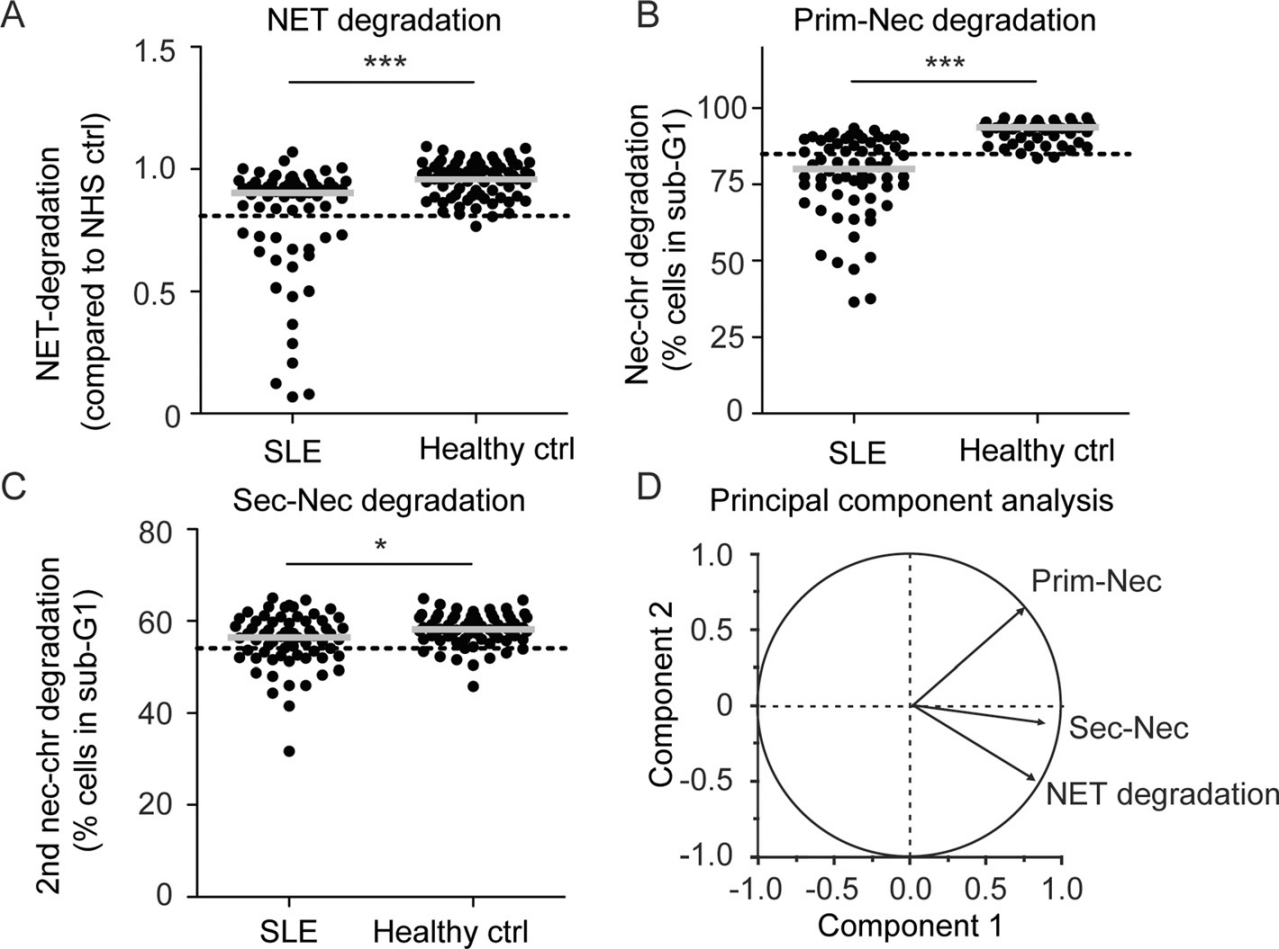
Sera from patients with systemic lupus erythematosus (*SLE*) had decreased ability to degrade multiple forms of chromatin. **a-c** Sera from 66 patients with SLE were used to determine degradation of neutrophil extracellular trap (*NET*) (**a**), primary (*Prim-Nec*) (**b**), and secondary (*Sec-Nec*) necrotic cell chromatin (**c**) in comparison to controls (*Healthy ctrl*) (**a**, *n =* 77; **b**, *n =* 50; **c**, *n =* 66). A cutoff below 2 SD below the mean degradation in the control group defined patients with low degrading ability and is indicated by the *dotted line*. **d** Principal component analysis for correlation revealed that degradation of secondary necrotic chromatin and NETs co-correlated. **a-c** Mean of three independent experiments with an average SD of 5 %. Significance of difference in (**a**-**c**) was calculated using the Mann-Whitney test; ***p <0.001; *p <0.05. *NHS* normal human serum

The ability of sera from 66 patients with SLE to degrade primary and secondary necrotic chromatin as well as NETs (the latter data were published previously elsewhere [17] but are used here for comparison), were investigated and compared to healthy controls. Throughout the study, we have defined low degradation as an activity of more than 2 SD below the mean of the healthy controls (Fig. 2, dotted line). In our titration experiments (Fig. 1) we noticed that degradation of secondary necrotic cells requires a higher serum concentration, therefore 3 % serum was used in this experiments compared to 1.5 % for degradation of primary necrotic cells. As previously shown, a subgroup of 30 % of serum samples from patients with SLE did not degrade NETs (Fig. 2a); interestingly a larger group of 62 % of SLE patients had decreased ability to degrade primary necrotic chromatin (Fig. 2b) and very few patients (14 %) were unable to degrade secondary necrotic cells (Fig. 2c). To determine how degradation of the chromatin sources correlated, we used principal component analysis. NET degradation was more closely correlated to degradation of secondary necrotic chromatin compared to primary necrotic chromatin (Fig. 2d). Next we investigated the role of serum nucleases for NETs and chromatin degradation.

### Degradation of chromatin from necrotic cells and NETs is carried out by DNase-I in complex with serum protein(s)

**Fig. 3:**
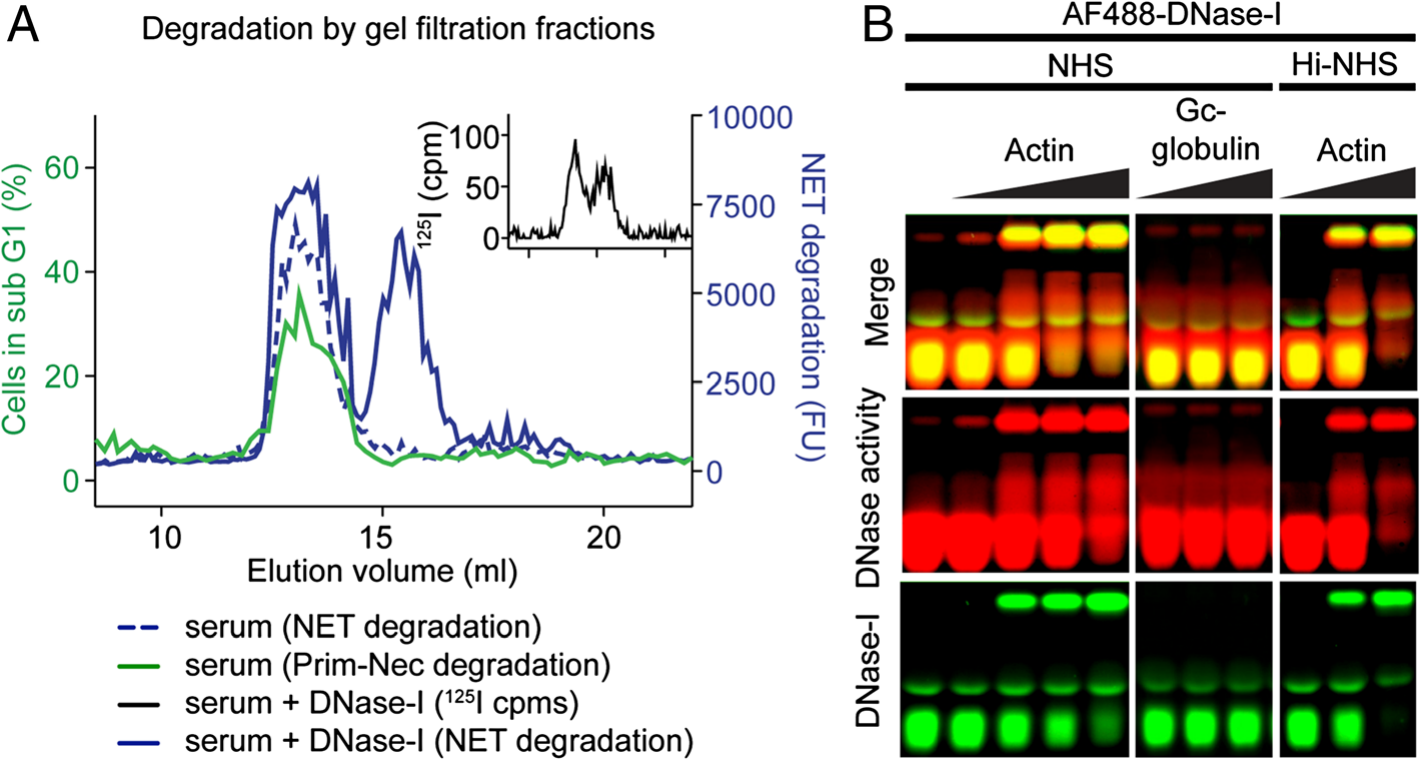
DNase-I binds to serum protein(s) but remains active. **a** Normal human serum (*NHS*) separated by size using gel filtration was tested for neutrophil extracellular trap (*NET*) degrading activity (*dashed blue line*, *right y-axis*) and primary necrotic chromatin degrading activity (*green line*, *left y-axis*). Activity was confined to one peak. Serum with ^125^I-labeled DNase-I was used to determine if degradation corresponded to DNase-I activity. The ^125^I DNase-I incubated with NHS eluted in two peaks as judged by ^125^I radiation (*insert*) indicating free DNase-I and DNase-I bound to a serum protein(s) or forming multimers. Proteins eluted in both peaks contained NET degradation activity (*solid blue line*, *right y-axis*). **b** AF488-labeled DNase-I was incubated with increasing concentrations of actin, or Gc globulin as negative control, in NHS or heat-inactivated NHS (*Hi-NHS*) and subjected to native DNA zymogram. At increasing concentrations of actin, DNase-I (*green*) shifted to a larger/less charged state although it remained active as judged by activity staining (*red*). **a** Representative data of two independent experiments. **b** Representative gel out of three independent experiments. *Prim-Nec* primary necrotic chromatin, cpm = counts per minute

It has previously been established that DNase-I is the major nuclease that degrades NETs [7] and necrotic chromatin [11]. To confirm the form of DNase-I serum activity we used size-separating gel filtration chromatography on NHS and used the size-fractionated serum to degrade NETs and chromatin from primary necrotic cells. We observed that the same fractions (around 13 ml) degraded both NETs (Fig. 3a, blue dotted line) and necrotic chromatin (Fig. 3a, green line). To confirm that this size corresponded to DNase-I we spiked NHS with radiolabeled DNase-I. Interestingly, we observed that radioactivity was eluted in two peaks (Fig. 3a, insert) and that eluted proteins of both peaks were able to degrade NETs (Fig. 3a, blue line). The second peak (around 16 ml) corresponded to the elution volume of monomeric DNase-I (data not shown). This indicates that active DNase-I is either in complex with a serum protein or in a multimeric form in serum. DNase-I binds to monomeric actin in serum, which is known as a strong DNase-I inhibitor [18, 19]. The results from our gel-filtration assay, however, suggest that DNase-I remains active. To test if this excludes actin as the DNase-I binding protein, we used a zymographic approach to test if actin indeed inactivates DNase-I in our setup. Using a native PAGE containing DNA and fluorescently labeled DNase-I to trace the protein and EtBr to detect loss of DNA in particular areas as a result of nuclease activity. By adding globular actin to serum spiked with DNase-I we observed that DNase-I bound to actin in a concentration-dependent manner (Fig. 3b). We also observed, as judged by the nuclease activity, that the DNase-I-actin complex remained active. As a negative control, no complex formation was observed using Gc-globulin, which binds the DNase-actin complex through actin [20]. Further, the actin-DNase-I complex also formed and remained active in Hi-NHS.

### DNase-I complex activity is not decreased in SLE patients

**Fig. 4:**
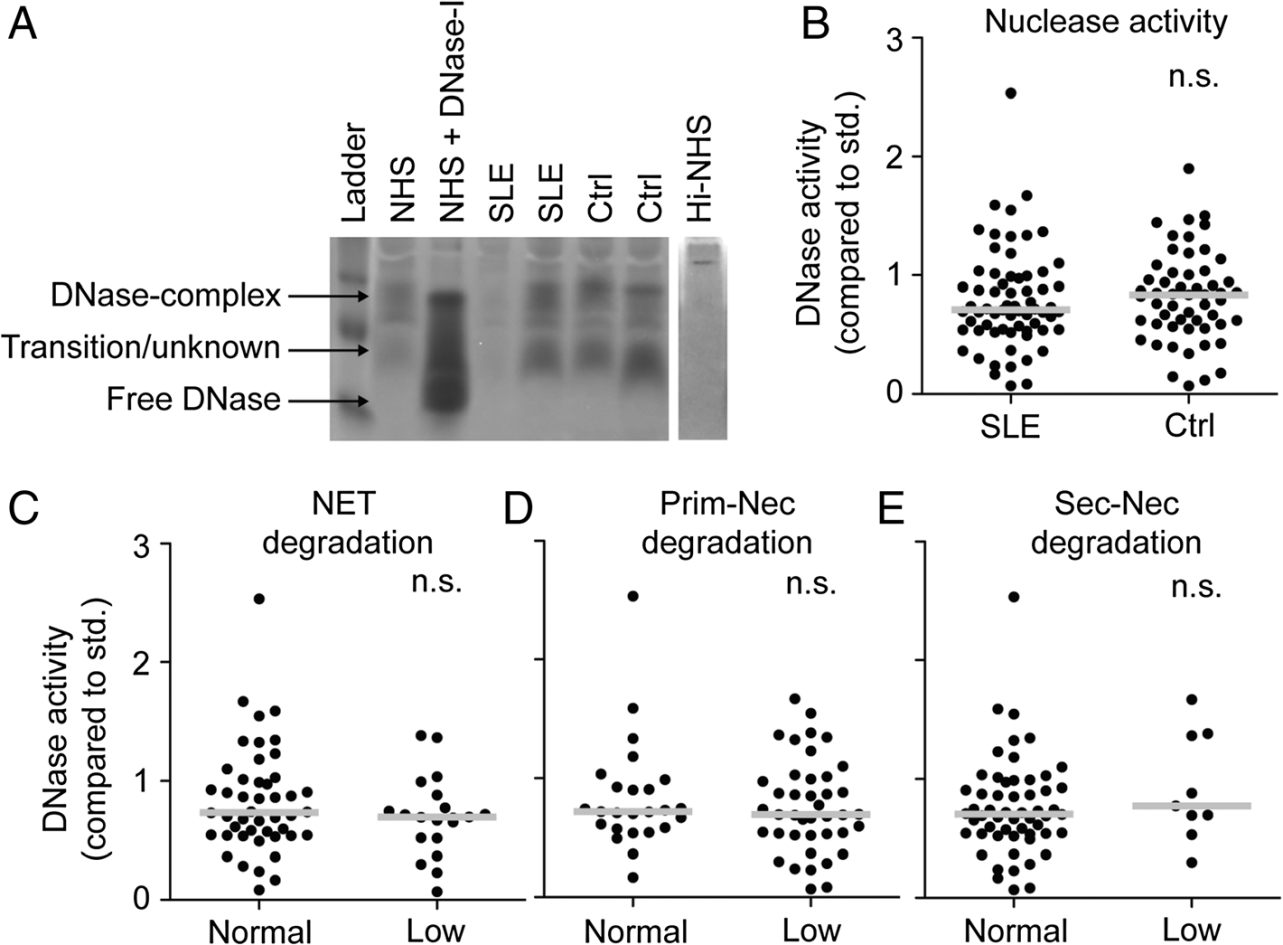
DNase-I complex activity does not differ in patients with systemic lupus erythematosus (*SLE*) compared to healthy controls. Normal human serum (*N* *HS*), heat-inactivated NHS (*Hi-NHS*) from SLE patients (n = 66) or sera from controls (*ctrl*) (n = 62) were separated on native DNA zymograms. Nuclease activity was detected as lack of ethidium bromide (EtBr) staining to DNA (*dark bands*). Heating efficiently inhibited all nuclease activity. **a** Representative zymogram for sera from two controls and two SLE patients with NHS as internal control and NHS + DNase-I to indicate free and bound DNase-I. **b** DNase-I complex activity was quantified and compared between SLE patients and controls. **c-e** Activity of the same band is shown for particular patient groups with low neutrophil extracellular trap (*NET*) degradation (**c**), low primary necrotic chromatin degradation (**d**) or low secondary necrotic chromatin degradation (**e**). Significance of difference was calculated using the Mann-Whitney test (**b**-**e**); *n.s.* not significant. *Prim-Nec* primary necrotic chromatin, *Sec-Nec* secondary necrotic chromatin, *std.* standard

After establishing that serum DNase-I is active as a protein complex in serum, we used the same zymographic approach to investigate if the activity of the complex is altered in SLE patients. In NHS we observed two bands of nuclease activity (Fig. 4a). As expected, these did not correlate with free DNase-I but resembled the bands induced by actin (Fig. 3b; top band represents the DNase-I-actin complex and the second a transition/unknown state). As our data suggest that the DNase-I-actin serum complex degrades NETs and necrotic chromatin, we used the top band to analyze serum nuclease activity. We discovered a large variation in activity between individual serum samples from both healthy controls and SLE patients with no significant difference between the two groups (Fig. 4a, b). Further, no difference was observed between SLE patients who did not degrade NETs, or primary or secondary necrotic chromatin (Fig. 4c-e). Together this demonstrates that although a large proportion of the patients in this SLE cohort have a decreased ability to degrade chromatin from particular cellular sources, when analyzed on pure and denatured DNA the activity of the serum DNase-I complex is not significantly decreased compared to healthy controls.

### Decreased degradation is associated with disease manifestation

**Fig. 5:**
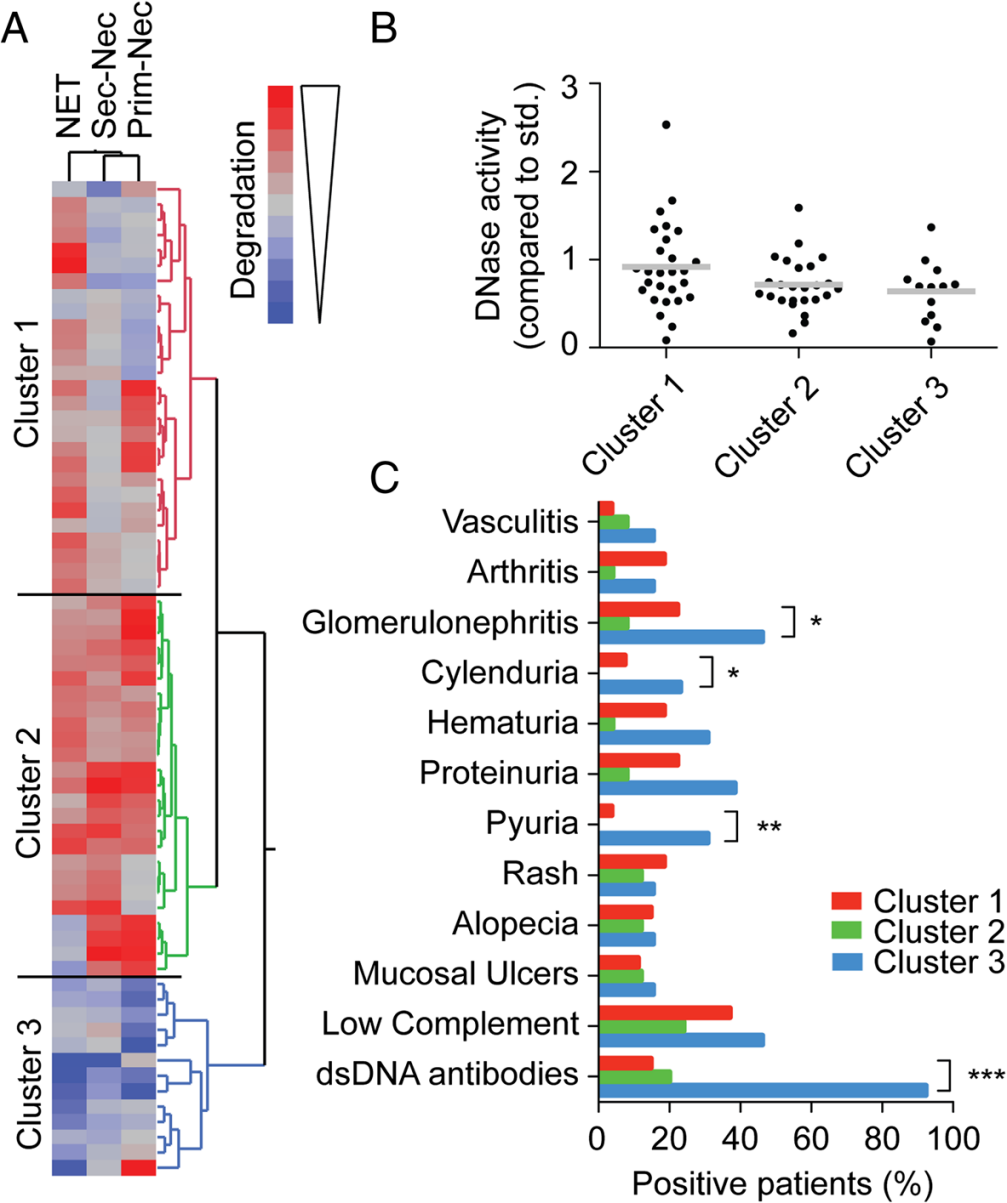
Degradation generates three distinct clusters of patients with distinct clinical manifestations. **a** Cluster analysis of degradation of neutrophil extracellular traps (*NET*s), primary necrotic and secondary necrotic chromatin displayed as *red* (normal) to *blue* (low) in the respective *columns*, one patient per *row* (n = 65). **b** DNase activity in the three clusters with no significant differences between the groups. **c** Proportions of patients with indicated systemic lupus erythematosus disease activity index manifestation in each cluster (only manifestations affecting >10 % of patients were included). Significance of differences was calculated using Pearson’s χ^2^ test; ***p <0.001; **p <0.01; *p <0.05. *Prim-Nec* primary necrotic chromatin, *deg* degradation, *std.* standard

We and others have previously observed that decreased ability to degrade NETs is associated with elevated titers of DNA antibodies and manifestations of glomerulonephritis [6, 7, 17]. To determine if this association is NET-specific or related to a general decrease in ability to degrade chromatin, we analyzed the distribution of SLEDAI qualifying manifestations in the groups with low and normal ability to degrade NETs, primary and secondary necrotic chromatin (Table 2). As previously established, decreased ability to degrade NETs was associated with glomerulonephritis and DNA antibodies. A similar pattern was also observed for patients who did not degrade secondary necrotic chromatin. However, patients who did not degrade primary necrotic chromatin only had an increased incidence of fever. To further isolate manifestations associated with a general decrease in DNA degradation in SLE we used cluster analysis based on the degradation data to classify patient subsets (Fig. 5a). The analysis rendered 3 clusters with cluster 1 including patients with a decrease in degradation of mainly primary necrotic chromatin. Cluster 2 included patients with mostly normal degradation, whereas cluster 3 included almost exclusively patients with a decreased ability to degrade all tested forms of DNA. Interestingly and as expected from previous results, we found that nuclease activity did not differ between the three clusters (Fig. 5b). When analyzing the presentation of clinical manifestations in the three clusters we found that manifestations associated with kidney disease as well as dsDNA antibodies were significantly more common in cluster 3 (Fig. 5c), suggesting these manifestations were associated with chromatin degradation independent of DNA source.

**Table 2:** SLEDAI-qualifying manifestations are associated with decreased ability to degrade chromatin from multiple sources

Manifestation	Total	NET	Primary necrotic chromatin	Secondary necrotic chromatin
		n = 20/46	n = 41/25	n = 9/56
		(% low/% normal)	(% low/% normal)	(% low/% normal)
Seizure	0	0/0	0/0	0/0
Psychosis	0	0/0	0/0	0/0
Organic brain syndrome	0	0/0	0/0	0/0
Visual disturbance	1	0/2.2	2.4/0	0/1.8
Lupus headache	0	0/0	0/0	0/0
Cerebrovascular accident	0	0/0	0/0	0/0
Vasculitis	5	15/4.4	4.9/12	11/7.2
Arthritis	8	10/13	12/12	11/13
Myositis	1	5/0	2.4/0	**11**/0*
Glomerulonephritis				
*Cylenduria*	5	15/4.4	9.8/4	11/7
*Hematuria*	10	20/13	17/12	33/13
*Proteinuria*	13	30/15	22/16	**44**/16*
*Pyuria*	**5**	**20**/2.2*	9.8/4	22/5.4
Rash	10	15/15	15/16	11/16
Alopecia	9	20/11	20/4	22/13
Mucosal ulcers	8	15/11	17/4	22/11
Pleuritis	3	5/4.4	4.9/4	11/3.6
Pericarditis	0	0/0	0/0	0/0
Low complement	23	35/35	39/28	56/30
dsDNA antibodies	**22**	**75**/15***	41/20	**67**/27*
Fever	4	5/6.5	**9.8**/0*	0/7.1
Thrombocytopenia	0	0/0	0/0	0/0
Leukopenia	4	5/6.5	4.9/8	0/7.1

Clinical manifestations during the time of sample collection were divided into groups according to their ability to degrade neutrophil extracellular traps (NETs), primary and secondary necrotic chromatin. Total patients with manifestation are indicated in the first column. For each source of DNA the total number of patients with low-versus-normal degrading ability are indicated and percent of patients with low-versus-normal degrading ability are indicated for each respective manifestation. Significance of differences were calculated using Pearson’s χ^2^ test: *p <0.05: and ***p <0.001; increased proportions in bold text

## Discussion

For unknown reasons the ability to degrade DNA is decreased in a proportion of patients with SLE. In this report we have systematically investigated the ability of sera from SLE patients to degrade DNA from multiple clinically relevant sources of DNA. We have focused the study on degradation of DNA sources that are dependent on serum nucleases as determined in previous reports [11, 15] and as shown in Fig. 1. Interestingly, the study reveals that the majority of patients with SLE (61 %, *n* = 40) exhibit decreased ability to degrade chromatin from either NETs, primary or secondary necrotic cells by serum. As there are multiple nucleases expressed in different tissues and cellular locations [21], it is tempting to speculate that studies of other means of degradation such as endogenous degradation of apoptotic chromatin by caspase-activated DNase [22] as well as other intracellular DNases such as TREX1 [23, 24] and DNase-gamma [25] may expand this group further and should be addressed in future studies.

Although there was relatively poor correlation between decreased degradation of different types of chromatin, we did identify a subset of patients with a distinct clinical phenotype associated with a general decrease in NET/chromatin degradation. Patients in cluster 1 mainly displayed a decreased degradation of primary necrotic chromatin, which points in a direction of a separate mechanism of degradation and aligns with previous reports describing cofactor-dependent degradation of the highly condensed chromatin from primary necrotic cells that require decondensation before efficient degradation. Presence of anti-DNA antibodies appears to prevent degradation of the decondensed NETs [7] and possibly also chromatin from secondary necrotic cells, which aligns with the clinical associations in cluster 3. Additionally, there are also reports of protein-DNA complexes, originating from NETs, being less efficiently degraded in SLE [26] which require further investigation in relation to other sources of DNA. To address the decreased ability to degrade primary necrotic chromatin in cluster 1, we used western blot to analyze protein levels of some DNase-I cofactors, such as C1q [8] and factor VII-activating protease [10]. We did observe a trend towards lower levels in particular of factor VII-activating protease in cluster 1, however this did not reach statistical significance (data not shown). Most likely, a more systematic approach employing more sensitive methods would be required to properly address this issue. The data in this study do suggest such study should focus on patients in cluster 1.

Mice that lack DNase-I develop SLE-like disease [27] and nephritis in mice and humans is also associated with a decrease in DNase-I expression [28]. Multiple studies have found decreased nuclease activity in patients with SLE [4, 29, 30]. However, these studies measured total nuclease activity using radial diffusion or oligonucleotide immune assays that neither separate forms of serum nucleases nor do they take into account the presence of potential nuclease inhibitors in serum. By using a zymographic approach, we separated serum proteins based on charge and therefore reduced the likelihood of interference with inhibitors that are not strongly bound to DNase-I. It is interesting to note that in this setting there was no difference in DNase-I activity between healthy controls and SLE patients, at least not in the investigated cohort. Even though DNase-I mutations in SLE are rare [31], investigation of such mutations or transcription forms in individuals with low activity may be of interest. The data do however suggest that it is not the DNase-I activity per se that is affected in these SLE patients but rather DNase-I is prevented from degrading chromatin, potentially by sterically blocking access for DNase-I by dsDNA antibodies generated during the disease. Additionally, we cannot rule out that patients with low ability to degrade primary necrotic chromatin produce lower levels of cofactors. Together, the data hence suggest that the decreased degradation observed in SLE is not a predisposing factor but rather a consequence of the autoimmune response most likely further fuelling disease pathology such as kidney disease. This aligns well with the pathology of the DNase^−/−^ mouse [32]. We recently found that an inability of SLE patients to degrade NETs is, in the majority of cases, not permanent but usually recovers within a period of 6 months indicating that this is a dynamic process that correlates with disease activity and levels of nuclear autoantibodies [17].

A pharmacological study in rats first observed that DNase-I binds a serum protein, which most likely is globular actin [18]. In the present study we can demonstrate that this complex is surprisingly active and appears to be the major form of DNase-I in the serum of healthy individuals. Inhibition of the actin-DNase-I complex is thought to be dependent on ATP, which was not included in our experimental setup. However when we used an ATP analog to confirm inhibition, disassociation of the complex occurred most likely due to polymerization of actin (data not shown). It is possible that this does not occur in vivo where there are actin depolymerizing factors and ATP stabilizing factors. Recently, this issue was addressed using gelsolin to prevent actin polymerization [33]. Intriguingly, as extracellular ATP is a marker of inflammation it is tempting to speculate that this may prevent NETs from being degraded at a site of inflammation and infection.

## Conclusions

The current study highlights the importance of the ability to properly degrade chromatin from multiple DNA sources in SLE and how such sources may influence clinical outcomes for the patient. We have identified a subset of patients with SLE with a decreased ability to degrade all forms of DNA/chromatin and this group poses a prime target for therapies aiming to increase degradation. However, our study also highlights the complex interactions of DNase-I and serum proteins that are important to consider in the future for the development of effective DNase-I therapy.

## Abbreviations

ACR: American college of rheumatology
ANOVA: analysis of variance
bp: base pairs
cpm: counts per minute
dsDNA: double stranded DNA
EtBr: ethidium bromide
Hi-NHS: heat-inactivated normal human serum
NET: neutrophil extracellular trap
NHS: normal human serum
PBS: phosphate-buffered saline
SLE: systemic lupus erythematosus
SLEDAI: systemic lupus erythematosus disease activity index
